# Computationally derived transition points across phases of clinical care

**DOI:** 10.1038/s41746-024-01145-1

**Published:** 2024-06-11

**Authors:** Aidan Gilson, David Chartash, R. Andrew Taylor, Laura C. Hart

**Affiliations:** 1 Department of Emergency Medicine, Yale University School of Medicine, New Haven, CT USA; 2 Section for Biomedical Informatics and Data Science, Yale University School of Medicine, New Haven, CT USA; 3 School of Medicine, University College Dublin - National University of, Ireland Dublin, Ireland; 4 Primary Care Pediatrics, Nationwide Children’s Hospital, Columbus, OH USA; 5 Departments of Internal Medicine and Pediatrics, The Ohio State University School of Medicine, Columbus, OH USA

**Keywords:** Epidemiology, Epidemiology, Health policy

## Abstract

The objective of this study is to use statistical techniques for the identification of transition points along the life course, aiming to identify fundamental changes in patient multimorbidity burden across phases of clinical care. This retrospective cohort analysis utilized 5.2 million patient encounters from 2013 to 2022, collected from a large academic institution and its affiliated hospitals. Structured information was systematically gathered for each encounter and three methodologies - clustering analysis, False Nearest Neighbor, and transitivity analysis - were employed to pinpoint transitions in patients’ clinical phase. Clustering analysis identified transition points at age 2, 17, 41, and 66, FNN at 4.27, 5.83, 5.85, 14.12, 20.62, 24.30, 25.10, 29.08, 33.12, 35.7, 38.69, 55.66, 70.03, and transitivity analysis at 7.27, 23.58, 29.04, 35.00, 61.29, 67.03, 77.11. Clustering analysis identified transition points that align with the current clinical gestalt of pediatric, adult, and geriatric phases of care. Notably, over half of the transition points identified by FNN and transitivity analysis were between ages 20 and 40, a population that is traditionally considered to be clinically homogeneous. Few transition points were identified between ages 3 and 17. Despite large social and developmental transition at those ages, the burden of multimorbidities may be consistent across the age range. Transition points derived through unsupervised machine learning approaches identify changes in the clinical phase that align with true differences in underlying multimorbidity burden. These transitions may be different from conventional pediatric and geriatric phases, which are often influenced by policy rather than clinical changes.

## Introduction

Multimorbidity, defined as the co-occurrence of multiple chronic conditions for an individual patient, is a fraught health challenge. Previous work has shown that for each additional morbidity, the risk of all cause mortality increased 23%^1^. The rising elderly population, alongside advancements in healthcare diagnostics and disease management, has resulted in a surge in people living with multimorbidity^2–4^. As a consequence, a deeper under-standing of multimorbidity patterns and transitions across different age groups is needed to address the complexities of healthcare delivery and ensure optimal patient outcomes.

Traditionally, age has been considered a key factor in determining disease prevalence and prognosis. However, the simple categorization of patients into broad age groups (e.g., children, adolescents, adults, older adults) overlooks potential nuanced variations within these cohorts^5,6^. Historically, these age cohorts were defined clinically and administratively as <18, 18–65, and 65 +. Although there are additional broad-based healthcare features that point to further stratification within these cohorts, i.e., neonates vs. children vs. adolescence, and 18–44 vs. 45–65, the delineation is less coherent, and these subcohorts are often grouped together for resource allocation. The selection of 18 and 65 as fundamental transitions between pediatric, adult, and geriatric care possesses some justification in medical gestalt; 18-year-olds may begin to present with more “adult” disease, while 65-year-olds present with more signs of chronic disease^7^. However, it can be argued that the driving force is more likely a legal definition centered around the transition to legal adulthood at 18, and federal policies around retirement age and Medicare enrollment averaging to 65 within the United States. These ages themselves are not intended to act as justification for seeing a pediatrician, adult generalist, or a geriatrician, rather they are a proxy for the implied differences in medical needs of the patients^8^.

By acknowledging that different age cohorts exhibit unique patterns of multimorbidity, healthcare professionals can optimize patient care, clinical decision-making, and resource allocation^9^. Furthermore, recognizing the distinct challenges and health needs within age-specific groups can contribute to the development of targeted interventions and preventive strategies^10,11^.

To improve our understanding of the unique needs of various age groups, a data-driven approach was used to identify phases of clinical care that may more closely align with the complex multi-morbidity differences that are present across the lifespan. This analysis would allow more effective planning efforts by health systems, payors, and policy-makers when considering health=care utilization, as the age groups would align with medical, rather than legal differences. To this end we present a quantitative approach to defining transition points in multimorbidity burdens across different ages. We leverage three methodologies: clustering-based, geometric reconstruction, and recurrence property-based phase space analysis; to identify both specific and general transition points in a cohort of multimorbid patients. By doing so, we aim to provide valuable insights on epidemiological cohorts, which describe the differences in multimorbidity across the lifespan in order to guide the optimization of patient care, clinical decision-making, and resource allocation.

## Results

### Cohort Definition

In total, a set of more than 5.2 million patient encounters were used in the analysis. These encounters were associated with 5,262,384 codes. Patient characteristics are shown in Table 1.

**Table 1:** Patient Characteristics of the Study Population

		Missing	Overall
n			5,229,969
Age, median [Q1,Q3]		0	41.8 [24.4, 60.2]
Gender, *n* (%)	Female	0	2,957,086 (56.5)
	Male		2,272,762 (43.5)
	Unknown		121 (0.0)
Race, *n* (%)	White or Caucasian	5382	2,794,837 (53.5)
	Black or African American		1,508,190 (28.9)
	Other		718,222 (13.7)
	Asian		97,370 (1.9)
	Unknown		69,697 (1.3)
	American Indian or Alaska Native		19,913 (0.4)
	Native Hawaiian or Other Pacific Islander		16,358 (0.3)
Ethnicity, *n* (%)	Hispanic or Latino	5442	1,178,045 (22.5)
	Non-Hispanic		4,015,922 (76.9)
	Unknown		30,560 (0.6)

### Diana

**Fig. 1:**
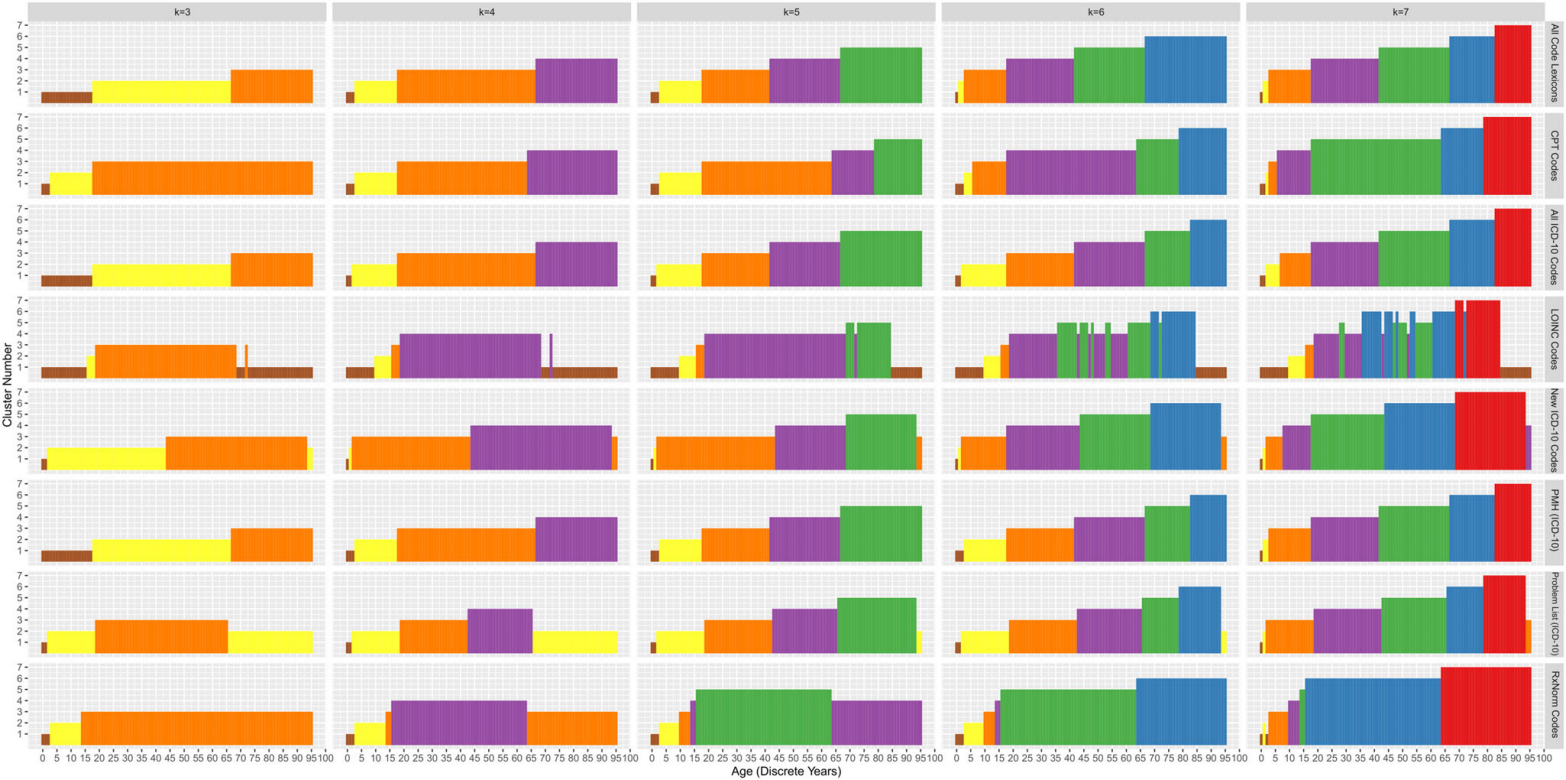
DIANA-based clustering across multiple lexicons and cluster numbers. The groupings of discrete ages for k-values 3-7 and different coding lexicons, including ICD-10CM (New Conditions, PMH, and Problem List), CPT, LOINC, RxNorm, and all combined.

The DIANA algorithm with k = 5 across all features achieved the highest silhouette score in the analysis, shown in Supplementary Fig. 1. Clustering results using DIANA for k of 3-7 on all codes, RxNorm, CPT, LOINC, new conditions, PMH, problem list, and all ICD-10 codes are shown in Fig. 1. With the exception of LOINC codes, each feature set led to clusters of sequential ages with only small groups of discontinuity. Exact age cutoffs of the clusters varied with k and the feature set, however for all features k = 5 the age groups were 0-2, 3-17, 18-41, 42-66, and 67-95. In contrast, k = 3 identified clusters with age ranges of 0-18, 18-68, and 68-95. While for k = 7 age groups of 0-1, 1-3, 3-18, 18-42, 42-62, 62-73, and 73-95 were identified. CPT codes had a large age range for patients in early to mid-adulthood, 17-61, with smaller clusters in the geriatric age range. The variation between new conditions, PMH, and a patient problem list was less than the variation between any two different terminologies. New conditions provided more stratification for very young patients, with a cluster of 0-1 and 1-2, grouping the remaining patients from 3-43 into one cluster.

### False nearest neighbors

**Fig. 2:**
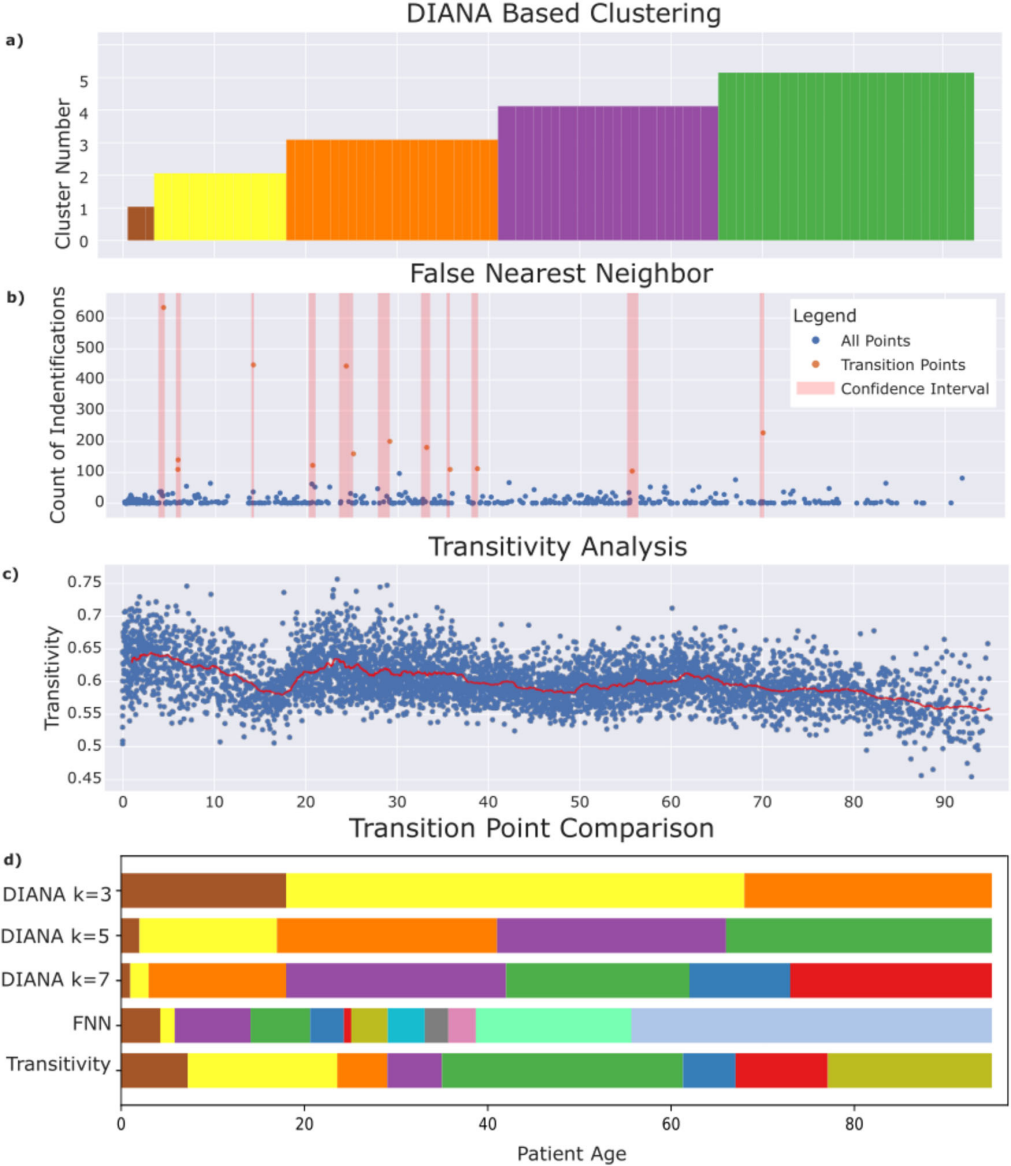
Comparison of Transition Point Identification Results. **a** DIANA Clustering with k = 5 (**b**) False Nearest Neighbor, transition points are shown in red with range of possible transition shown as a red bar (**c**) Transitivity analysis of patient age ordered encounter embeddings. **d** Comparison of DIANA (k = 3,5,7), FNN, and Transitivity based age groups.

The results of the OPERAND methodology are shown in Fig. 2(b). Rather than identifying complete clusters, this method computes transition points in the phase space (embedding dimension) of the age-ordered patients. These transition points represent the change in the phase space such that the number of false nearest neighbors are minimized, therefore identifying a shift from one temporally identifiable stage of health to another. A transition point can therefore be thought of as a change between patient-age cohorts. In total, 13 transition points were identified at ages 4.27, 5.83, 5.85, 14.12, 20.62, 24.30, 25.10, 29.08, 33.12, 35.7, 38.69, 55.66, 70.03. This resulted in 11 zones of transition as the range of possible transition of ages 5.83 and 5.85 as well as 24.30 and 25.10 were overlapping. A common transition point was selected as the average of the two combined points. Leading to final transitions at 5.84 and 24.70.

Supplementary Fig. 3 shows the running average of the total transition points found in a one-year window surrounding each age. Rather than a single point of transition, this represents the overall variation in the phase space as represented by the total number of times a change in the embedding dimension was found to be significant.

### Transitivity

The results of the transitivity analysis are shown in Fig. 2(c). To evaluate the potential transition points, we evaluated the coefficient of variance D_T_ across a running range of 100 windows consisting of 1000 patients each.

Transition points from all methods are shown for comparison in Table 2.

**Table 2:** Transition points detected by each method

Methodology	Transition points
Diana k = 3	18, 68
Diana k = 5	2, 17, 41, 66
Diana k = 7	1, 3, 18, 42, 62, 73
False nearest neighbors	4.27, 5.83, 5.85, 14.12, 20.62, 24.30, 25.10, 29.08, 33.12, 35.7, 38.69, 55.66, 70.03
Transitivity analysis	7.27, 23.58, 29.04, 35.00, 61.29, 67.03, 77.11

## Discussion

In this large-scale retrospective study of more than 5.2 million individual patient encounters, we investigated three different approaches for the identification of transition points between stages of patient care: DIANA-based clustering of patient ages, the OPERAND method based on FNN analysis by Neuman et al., and transitivity analysis derived from recurrence plots in the phase space of patient embeddings^12^. These methods represent a range of approaches examining patients at the individual and group level. Across all methodologies, we found that there was some alignment to pre-established age transitions, giving validation to conventional pediatric/geriatric age cutoffs. However, we found that a greater number of transition points was needed to divide the entire age-ordered population into like-cohorts. More specifically, we found that there were more transitions in the 20–40 age range. A potential interpretation of this result is that the age range of 20-40 is where many patients develop a first chronic diagnosis, or where earlier health events or choices lead to downstream outcomes. Conversely, while numerous transition points were found in the 20-40 age range, a range considered clinically homogeneous, few points were found between ages 3-17, a range often considered more heterogeneous. Although there is significant social and developmental changes in this age range, their structured clinical presentation may be consistent as a result of a minimal multimorbidity burden at that age range.

Examining first the similarity to conventional transitions, even with the precision offered by these methods, there was a striking resemblance to the clinical gestalt or physicians’ expectations. For instance DIANA based clustering at k = 3 led to clusters that roughly match the ages that delineate pediatric and adult as well as adult and geriatric care. However, the silhouette-metric optimal clustering performance occurred with five clusters. The groups, 0–2, 3–17, 18–41, 42–66, and 67–95 represent ages of infancy/toddler, childhood/adolescence, early adulthood, late adulthood, and geriatric populations. This may indicate that current clinical parameters overvalue certain age delineations, i.e. the difference between ages 2-11 as childhood and 11-18 as adolescence^13^. Current clinical breakdowns may also undervalue further stratification in other age ranges, for example, the difference between early adulthood and late adulthood.

However, the age groups identified through cluster-based analysis are not consistent across the different feature sets: ICD-10CM, CPT, LOINC, and RxNORM. For example, the age breakdowns of ICD-10CM codes are similar to all features together, while CPT-based clustering with k = 5 was 0–3, 3–18, 18–64, and 64–78, 78–95. This breakdown groups all adults into one cluster while providing more stratification in the geriatric population. It is difficult to elucidate the exact reason for the difference; however, one hypothesis might be the increased rates of screening procedures that a younger geriatric population receives, while at 75 most screening procedures are suspended while procedures become more diagnosis specific.

By employing the OPERAND methodology, we are able to investigate transition points with greater granularity than a one-year interval, and range of possible transition of these points may be interpreted as potential “transition zones.” These transition zones represent clinical heterogeneity at the individual and population levels. It is unreasonable to expect every patient to transition between clinical phases at a precise age, and it is also not accurate to say that any one patient suddenly transitions at a specific age, whatever that age may be.

The OPERAND-derived age groups shed light on the potential oversimplification of treating all adult patients as a single cohort. Notably, these transitions predominantly revolve around the 20–40-year-old age range as stated previously, which is conventionally viewed as clinically homogeneous. As show in in Fig. 2(d), three transition zones: around ages 4.25, 5.8, and 14 occur in the pediatric age range. While two transition zones around ages 55.6 and 70 occur in the older adult phase, and the remaining six: ages 20.6, 24.75, 29.1, 33.1, 35.7, and 38.7 occur in only an 18-year window. As noted earlier, these transition points may signify the onset of chronic diseases in patients. Patients tend to exhibit fewer associated codes for their care at younger ages, indicating a period of relative health, while at older ages they tend to manifest a stable heterogeneity, indicating diverse health outcomes. Although older patients may not be similar, they have reached various health outcomes. The ages of 20–40 encompass the overall transition between these stages.

Supplementary Fig. 3 provides another mechanism for interpreting the OPERAND results. Through the use of a 1-year running sum, the graph approximates the overall perturbations in the phase space of the patients within that window. Three peaks are defined around the ages 4, 14, and 24. Although all transition points should be considered changes between clinical phases of patients, the magnitude of the peaks in the 1-year window could be interpreted as the degree of change between phases before and after the peak. For example, the transition of the stage of healthcare before and after 24 years of age may be of greater difference than before and after 70, which has a lower peak.

Finally, we use transitivity analysis on the same phase-space representation. Figure 2c shows the DT of groups of 1000 patients ordered by age. Although transition points are not identified, this graph may represent the flux, or how much change is occurring, in the phase space at that age range. For instance, between ages 18 and 22 there is a significant increase in DT, which may indicate a large transition in the nature of patient complexity at that time interval. Supplementary Fig. 2 shows the coefficient of variance of DT. Peaks of this variance represent areas with higher than average variation in DT at that age. These peaks may be interpreted as transition points identified through the transitivity analysis.

A limitation of this analysis is the self-fulfilling nature of the current clinical gestalt that the analysis may be identifying. The reason some of the transition points identified are similar to current clinical age cutoffs may be because physicians are influenced in the diagnoses, labs, medications, and procedures these patients are receiving when they cross these age cutoffs. For example, was a transition point at 18 years old identified because there truly is a change in the stage of patient care independent of current clinical gestalt, or was it found because patients are already changing from pediatric to adult providers at that time, and our analysis is simply identifying that change. Validation in a dataset of patients from other institutions would help confirm these results. The methodology employed in this analysis offers a high level of reproducibility, largely owing to its reliance on established methodologies and readily available tools. The foundation of our approach rests heavily on the work of Neuman et al., who have provided comprehensive Python and MATLAB packages for both the OPERAND and transitivity-based methodologies^12^. Furthermore, the Diana-based clustering can be executed using numerous pre-existing packages. Additionally, all structured data was obtained through the Observational Medical Outcomes Partnership (OMOP) Common Data Model created by the Observational Health Data Sciences and Informatics (OHDSI) program^14^. Facilitation by OHDSI would allow for the analysis of a large-scale multi-institutional dataset on the previously described existing pipelines.

Our analysis has proposed three methods to investigate the stages of patient care in the healthcare setting. Our results indicate that current clinical gestalt may be similar to transition points identified through unsupervised approaches. An important distinction is that through all analysis methods, we identified multiple subgroups within the “adult” population conventionally thought of as 18–65, which are often treated as one patient population. This heterogeneity in the adult population may be a starting point for more granular consideration of age-specific care needs among adults. Furthermore, it may provide insight into resource allocation and efforts for when interventions before the development of new diseases in a patient may be most efficacious. More testing is needed to examine the clinical and epidemiological potential and validity of these transition points.

## Methods

All analysis was conducted in Python 3.10.5. This research was deemed exempt under 45 CFR 46.104 (4) by the Human Research Protection Program of Yale University (HIC# 2000031389).

### Cohort creation

This was a retrospective observational cohort study of all primary care and hospital visits between 2013 and 2022. All patients with at least one encounter in the healthcare system were included in the analysis. The study sites were an academic urban medical center and an urban safety net medical center in the northeastern United States. These two facilities cover the second largest population center in their state of coverage. The region covered by this healthcare network has been consistently evaluated to be demographically similar to the composition of the national population^15–17^.

### Data collection

**Fig. 3:**
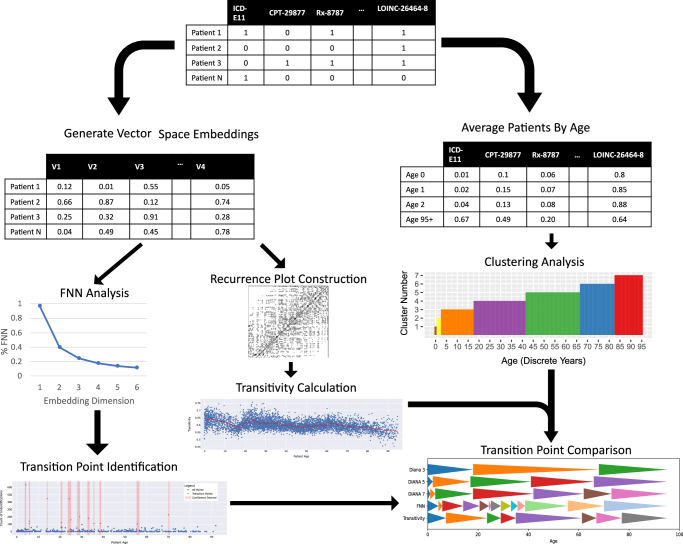
Summarization of Methods. Three methods of analysis used to generate novel transition points. FNN analysis and Transitivity analysis are performed on patient vector embeddings. Clustering analysis is performed on average rates of feature prevalence in each age group.

For each encounter in the electronic health record, we collected patient demographics, including age, sex, patient self-reported race, and date of death if the patient was deceased. For all patients with complete demographic information across age, sex, and race, we then collected the medical, procedural, medication, and laboratory codes associated with the patient. Medical and surgical information was extracted in the form of ICD-10CM diagnostic codes. ICD-10CM codes were classified into three categories: past medical history (PMH), represented by any code present before the beginning of the encounter; problem list, defined as any code which was present prior to the beginning of the encounter, and which was still currently an active diagnosis; and new conditions, defined as any code applied to the patient during the encounter. Each ICD-10CM diagnostic code was pared to a three-character category within a chapter and block (i.e., E11.11 - Type 2 diabetes mellitus with ketoacidosis with coma becomes E11 - Type 2 diabetes mellitus within Chapter IV Endocrine, nutritional and metabolic diseases, and block E10- E14 diabetes mellitus). Current Procedural Terminology (CPT) codes were used to identify procedures that the patient received and any procedure performed during the encounter. RxNorm codes were used to identify active medications in patients. Laboratory tests during the encounter were represented by Logical Observation Identifier Names and Codes (LOINC) codes. Each patient encounter was therefore represented by an age, sex, race, and a set of ICD-10CM, CPT, RxNorm, and LOINC codes. Subsequently, we employed three methodologies to investigate the characteristics of this data: a clustering-based approach, a geometric method based on the false nearest neighbor algorithm, and transitivity-based analysis of the phase space of the patients. A graphical representation of these methods and their comparison is presented in Fig. 3.

### Clustering analysis

First, we use multiple clustering algorithms to group patients by age. The age of the patients was rounded to the nearest integer value at the time of encounter. For each age among all patients in that age, we recorded the total number of encounters with each ICD-10CM code (separated into PMH, problem list, and new conditions), CPT, RxNorm, and LOINC codes. RxNorm codes were aggregated as RxClass values using the Anatomic Therapeutic Chemical Classification System (ATC) class system^18^. Patients older than 95 years were excluded from the analysis due to the notably lower sample size within that age bracket.

**Fig. 4:**
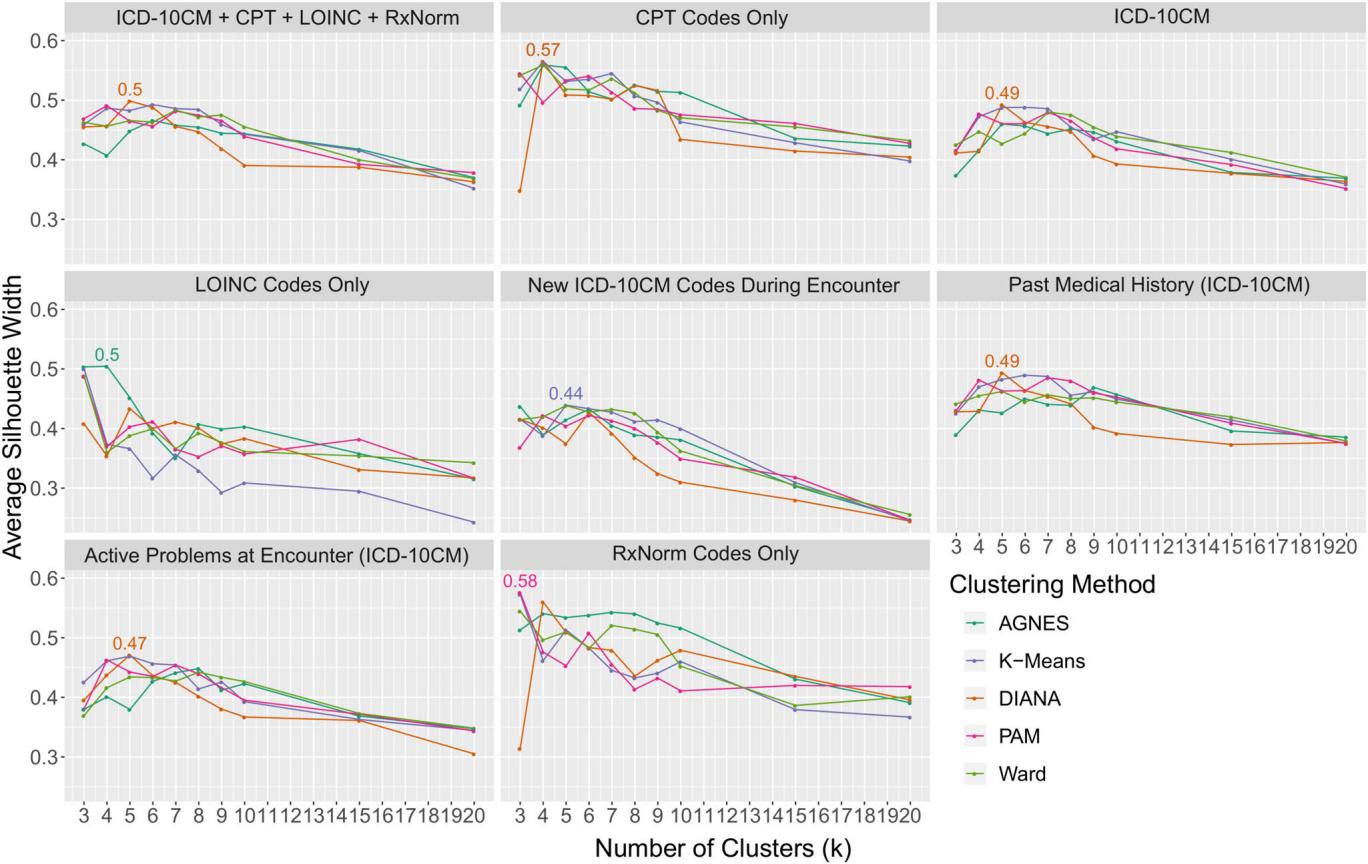
Silhouette Score by clustering method, coding lexicon(s) used, and number of clusters. The highest silhouette score across all cluster numbers and algorithms is shown for each coding lexicon. Note highest score is achieved with DIANA-based clustering.

K-Means, DIANA, AGNES, PAM, and agglomerative clustering with Ward’s minimum variance method were performed on the 95 age groups^19^. To account for the inherent variability of the data, we selected these algorithms to cover a variety of types of clustering techniques: hierarchical, k-mediods, k-means, and agglomerative algorithms, to empirically select for optimum performance. We performed the clustering on each coding set individually as a feature list of the age groups, as well as the combination of all coding sets. Cluster coherence was evaluated through silhouette scores and the DIANA algorithm was selected as the optimal technique, shown in Fig. 4.

### Constructing phase space - feature embedding

Next, we constructed vector embeddings for each encounter based on the features present in the coding sets. Each encounter was represented by n-features where each feature was a ICD-10CM, CPT, RxNorm, or LOINC code. We constructed a term co-occurrence matrix, and singleton counts matrix by recording the pairwise combinations of all codes across all individual encounters. We then applied the word2vec embedding model on these matrices to create 256-dimensional vector representations for each code. A vector representation for the encounter could then be constructed by taking the average embedding of the individual embeddings represented by all the codes in each encounter. It has previously been shown that the arithmetic mean of source embeddings often outperforms the construction of complex meta-embeddings with novel models^20^. The ordered embeddings of the encounters by patient age at the time of the encounter then represents a phase space of clinical progression. We use this phase space to follow the Optimal Embedding tRANsition Detection (OPERAND) method as well as the recurrence property method (detecting a change in transitivity coefficient of the phase space), both outlined by Neuman et al. ^12^.

### Geometric reconstruction of phase space - false nearest neighbor algorithm

The OPERAND methodology is described in detail by Neuman et al. ^12^. and the implementation used in more detail in the supplements. In brief, the identification of transition points in the phase space using false nearest neighbors (FNN) is parameterized using three variables: the window size of patients, the step size between windows, and the slope between adjacent embedding dimensions that was considered to be a significant change. We constructed a grid search to identify the most frequent transition points independent of the specific parameterization of said variables. First, the number of patients included in the adjacent windows by age was varied between 1000 and 10,000 patients in 1000-patient intervals. The threshold for the percentage of FNN in the embedding dimension for each window was set to 0.1. Next, the change between the embedding dimensions of the adjacent windows were examined. This change was calculated as the slope between both two adjacent windows and three adjacent windows to capture shorter and longer transition intervals allowing for the tuning of two parameters. A grid search was conducted, exploring the range of slopes considered significant in the change of embedding dimensions, ranging from differences between two to thirty across adjacent windows.

Each combination of window size, the significant slope for pairwise windows, and significant slope for triplicate windows was computed and the transition points for each recorded. The weight of each transition point was proportional to 1/n where n is the number of transition points identified by that combination of parameters. Transition points were said to be significant if they received a cumulative weight of 100 across all combinations of parameters. This indicates that they are identified across a range parameterizations, and are therefore less sensitive to the manual selection of specific parameters. We defined the range of possible transition around a transition point as follows. We first identified the average count of all transition points which were identified at least one time. We then defined the lower end of the range of possible transition to be the transition point closest to but with a lower age than the significant transition point with a count less than or equal to the average count of all transition points. The upper bound was defined similarly as the interval to the transition point closest to the significant point with a greater age that meets the same criteria.

### Recurrence property of phase space - transitivity analysis

Finally, using the same constructed phase space we performed transitivity analysis on the recurrence plots computed for each patient, again previously described by Neuman et al. ^12^. Similarly to the OPERAND methodology, a dimensionality measure is calculated, called ${D}_{{\mathcal{T}}}$ for this analysis. This measure is constructed from the geometric properties of the recurrence plot of the phase space for windows of patient encounters. The dimension measure ${D}_{{\mathcal{T}}}$ can then be compared across windows, just as the optimal embedding dimension from the OPERAND method is compared across adjacent windows. We used an embedding dimension of 3, recurrence threshold of 0.7, and window size of 1,000 patients following guidelines outlines by Neuman et al. For each window of patients, the transitivity coefficient ${\mathcal{T}}$ is calculated. The dimensionality measure ${D}_{{\mathcal{T}}}$ is then calculated as $\frac{{\mathcal{T}}}{\mathrm{ln}(3/4)}$. Both the absolute value ${D}_{{\mathcal{T}}}$, as well as the coefficient of variance of ${D}_{{\mathcal{T}}}$ across a 100 window average were calculated.

### Supplementary information

Supplementary Material

## Data Availability

Primary data is unavailable due to HIPAA compliance. For information on obtaining a minimum de-identified dataset for reproduction please contact the Yale IRB at irb.support@yale.edu.

## Code availability

All analysis was conducted in Python 3.10.5.Code will be made available at https://cchteam.med.yale.edu/gitlab/.
