# Supplementary material for: Computationally derived transition points across phases of clinical care
Source: NPJ Digit Med. 2024 Jun 11;7:151. doi: 10.1038/s41746-024-01145-1 (PMC11167560; doi:10.1038/s41746-024-01145-1)
Supplement: Supplementary file 1 — Supplementary Material [file 41746_2024_1145_MOESM1_ESM.pdf]

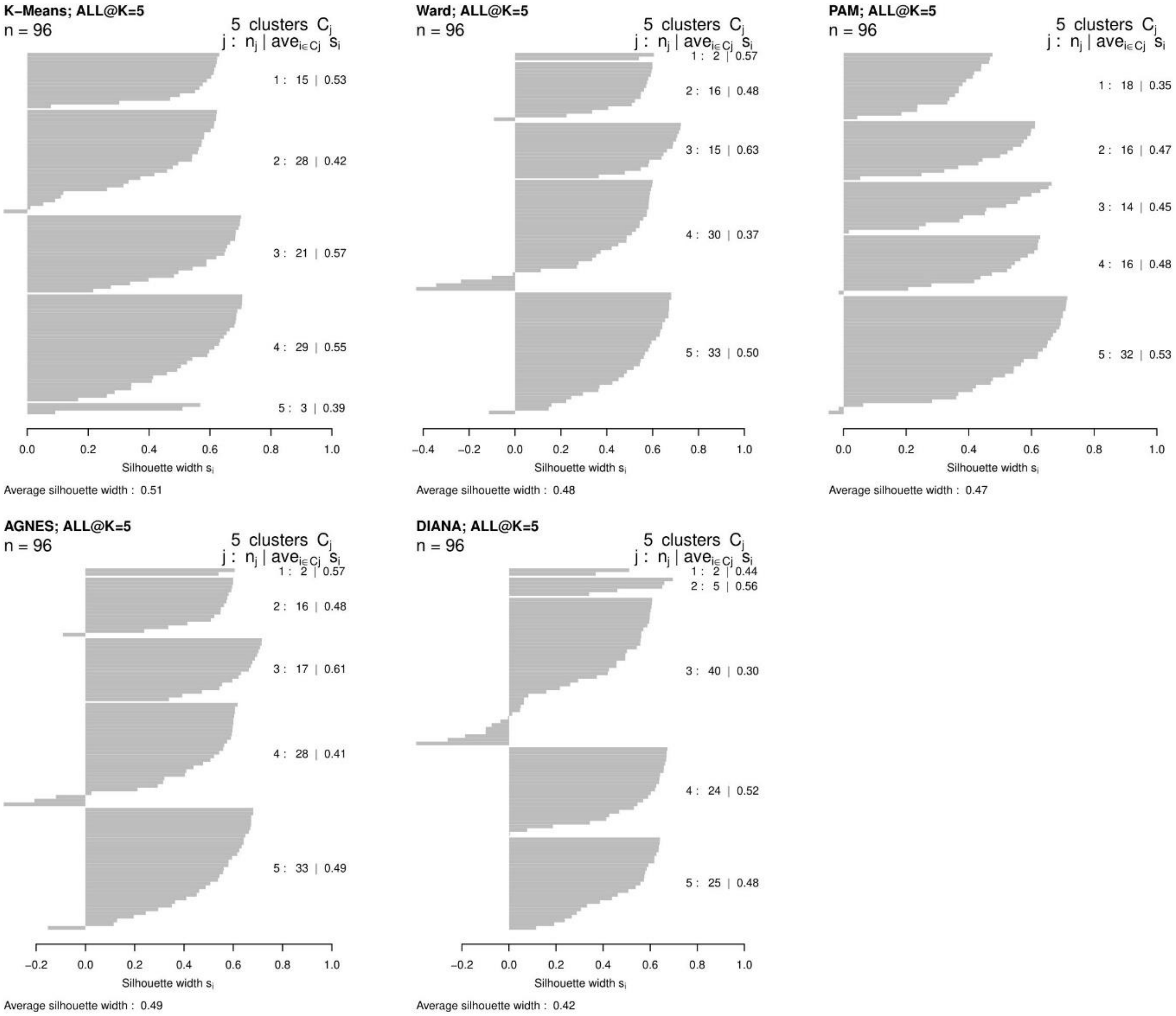

Supplementary Figure 1: Silhouette Scores of various clustering methodologies with k=5.

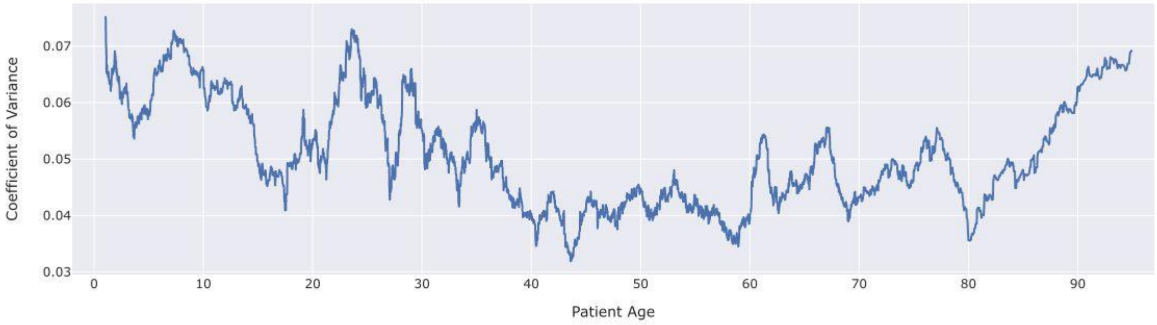

Supplementary Figure 2: Running Window of Coefficient of Variance

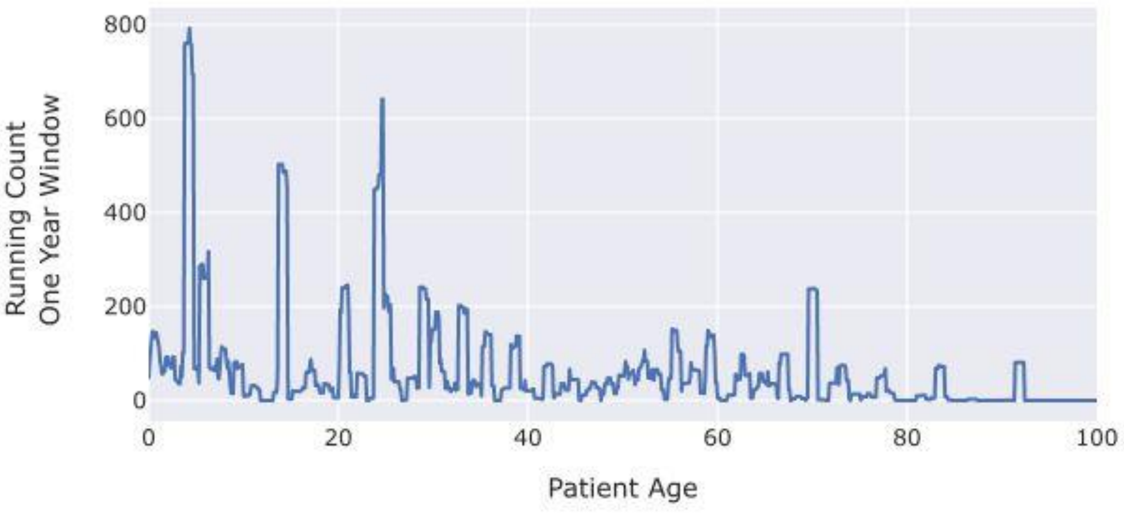

Supplementary Figure 3: Running average of Transition Points
